# Comparative evaluation of RBPT, I-ELISA, and CFT for the diagnosis of brucellosis and PCR detection of Brucella species from Ethiopian sheep, goats, and cattle sera

**DOI:** 10.1186/s12866-023-02962-2

**Published:** 2023-08-10

**Authors:** Abinet Legesse, Aregitu Mekuriaw, Esayas Gelaye, Takele Abayneh, Belayneh Getachew, Wubet Weldemedhin, Takele Tesgera, Getaw Deresse, Kenaw Birhanu

**Affiliations:** 1 Research and Development Directorate, National Veterinary Institute, P.O. Box 19, Bishoftu, Ethiopia; 2 Food and Agriculture Organization of the United Nations, Sub-Regional Office for Eastern Africa, P.O. Box 5536, Addis Ababa, Ethiopia

**Keywords:** Brucella species, Diagnosis, CFT, I-ELISA, RBPT, PCR

## Abstract

**Background:**

Brucellosis is an economically devastating animal disease and has public health concern. Serological methods such as Rose Bengal Plate Test (RBPT), Complement Fixation Test (CFT), and Indirect-Enzyme-Linked Immunosorbent Assay (I-ELISA) have been used to detect brucellosis. However, there is limited comparative evaluation studies and lack of molecular confirmation of the causative agents in the study areas. The study was aimed to compare RBPT, I-ELISA, CFT, and confirmation using Polymerase Chain Reaction (PCR). A total of 2317 sera samples were collected from brucellosis-affected areas of Ethiopia with no vaccination history. All sera were subjected to comparative serological assays. Post-cross tabulation, sensitivity, and specificity were determined using Receiver Operating Characteristics (ROC) curve analysis software. PCR was performed on 54 seropositive samples using genus- and species-specific primers.

**Results:**

Among the 2317 sera tested for comparative serological assays, 189 (8.16%) were positive for RBPT, 191 (8.24%) for I-ELISA, and 48 (2.07%) for CFT. Sensitivity to RBPT was 100% (95%) in shoats and 74% (95%) in cattle. Specificity on RBPT was 98.69% (95%), 99.28% (95%), 100% (95%) in sheep, goats, and cattle, respectively. CFT sensitivity was 4 (95%) in sheep, 9.65 (95%) goats, and 72 (95%) cattle. Specificity on CFT was 100% (95%) for sheep, goats, and cattle. A 223bp Brucella genus-specific and 156bp *B. abortus* species-specific detected. However, *B. melitensis* not detected.

**Conclusion:**

In this study, I-ELISA was the most sensitive and specific test. RBPT detected all Brucellosis-infected sheep and goats; nevertheless, it showed false positive in sheep and goats and false negative in cattle. The presence of *B. abortus* in small and large ruminants was confirmed by PCR. This is the first report of *B. abortus* detection in small ruminant in Ethiopia. *B.abortus* detected in non-preferred hosts. The findings suggest further study on molecular epidemiology of Brucella species.

## Introduction

Brucellosis is caused by the genus Brucella, a facultative intracellular and gram negative Pathogen responsible for major economic burden in livestock and human health concerns worldwide [1]. Brucellosis is a serious zoonotic infection [2] that is wide spread in Central and South America, Mediterranean European countries, India, Central Asia, Near East countries, Northern and Eastern Africa, and Mexico [3]. It is also thought to be a recurring problem in many countries, such as Colombia, Arabia, Brazil, Kuwait, and Israel. Increased calving intervals, decreased milk production, abortion, retained placenta, birth of weak and dead offspring, and infertility are all financial consequences of Brucellosis [4]. It is primarily a sexually matured animal disease, and animal-to-animal transmission is commonly through direct and indirect contacts. Discharge such as fluids, placental sheaths, and aborted fetuses can all be sources of infection [2, 5]. Human infection is caused by direct contact with diseased animals; the accidental injection of live vaccines; and the consumption of Brucella-contaminated raw milk and other unpasteurized dairy products [2, 3, 6].

Brucellosis was first reported in Ethiopia in the 1970s [7]. Based on brucellosis sero-prevalence studies conducted by several researchers, bovine brucellosis prevalence reported in various areas of the country. For example, 4.21% prevalence from local cattle in the central highlands [8]; 10.6% farm level sero-prevalence status of bovine brucellosis in Ethiopia [9]; 11% in traditional management practices [10]; 1.66% and 13.7% individual and herd level, respectively in southern Ethiopia (Sidama Zone) [11]; and 13.6% and 2.9% in three agro-ecological areas of central Oromia [12] were reported.

According to Yohannes and his colleagues [13], there is a scarcity of information on Ethiopian small ruminant brucellosis. However, here are some findings: Ashenafi et al. [14] conducted a cross-sectional study on 1005 goat and 563 sheep sera in the Afar region and found 4.8% by CFT and 9.4% using RBPT sero-prevalence of small ruminant brucellosis. Small ruminant brucellosis sero-prevalence studies were also conducted in Oromia, South Nation Nationslity People (SNNP), and Eastern and North Western Amhara regions of Ethiopia [15, 16]. Using CFT and RBPT, they found 0.4–4.89% sero-prevalence. All cited and visited authors only conducted sero-prevalence studies using RBPT and/or CFT. Among those findings, none of them made any attempt to isolate the brucellosis-causing etiological agent that was circulating in their study areas [13].

Brucella species have a preference for certain hosts. *Brucella abortus* is the most pathogenic and is responsible for cattle brucellosis [17], but *Brucella suis* and *Brucella melitensis* can also cause disease in cattle [18]. *Brucella melitensis* is the most common cause of brucellosis in sheep and goats [17]. Although *B. ovis* infects sheep, it is not thought to infect humans. *Brucella suis* is the primary cause of brucellosis in pigs. Isolation of Brucella from body fluids, exudates, blood, and tissues is absolute evidence for infection and has a 100% test specificity, but detection of cultures decreased with chronic infection [19]. In chronically infected animals, the outcome of bacterial cultures unusually low. This is usually the case when reaching an unquestionable conclusion is both challenging and essential. Brucella in serum and blood specimens have been identified using molecular techniques. Polymerase Chain Reaction (PCR) can identify a very small amount of bacteria in a specimen and is thus widely used method to detect brucellosis. However, according to different reports, its sensitivity ranges from 50 to 100% [20–22]. Some findings have revealed that serum samples are superior to blood specimens and can improve PCR sensitivity [23].

Classical serological tests, such as tube agglutination and RBPT, have been shown to produce non-specific reactions with other Gram-negative bacteria that have antigenic matches to Brucella [24]. To avoid such non-specific reactions, ELISA tests have been used for brucellosis detection. The superiority of ELISA tests stems from the use of different antigen preparations, anti-globulin enzyme conjugates, and substrates. The I-ELISA test successfully distinguished Brucella antibodies from other cross-reacting bacteria such as *Yersinia enterocolitica* serotype O:9 [24–27]. Despite the drawbacks mentioned above, serological tests remain an important diagnostic tool for brucellosis in endemic countries. In general, serology has maintained its scientific application and acceptance in the diagnosis of infectious diseases. In comparison to culturing or nucleic acid amplification methods, it is inexpensive and technically simple.

In Ethiopia, there is little or no genotyping of Brucella species or comparative serological assay study for brucellosis diagnosis. Comparative evaluations of test methods are required to conduct an accurate diagnosis of livestock brucellosis, to address appropriate detection methods for further brucellosis prevention and control. Therefore, the present study was conducted to evaluate the brucellosis diagnostic capacity and discriminative power of three serological tests: RBPT, CFT, and I-ELISA, as well as antigen detection of Brucella species using PCR in sera from sheep, goats, and cattle Ethiopia.

## Materials and methods

### Study areas, animals, and sample collection

Serum samples were collected from brucellosis affected areas in Ethiopia between December 2013 and November 2018. These include; the Oromia region (Jimma, Bale, Debre-Zeit, and Metehara), Amhara region (North Western Ethiopia and North Eastern parts of Amhara region), Afar region (Worer and Awash), and South Nation Nationality People region (Mizan and Dasenech). The animals had been kept under extensive management practices and with no history of Brucella vaccination. Approximately 10 ml of blood was collected from the jugular vein of sheep ( n = 552 ), goats (n = 1345 ) and cattle ( n = 420 ) using plain vacutainer tubes. The samples were left at room temperature overnight to allow clotting for serum separation as previously described by [12]. The serum was collected and stored at -20 °C until tests were performed at the National Veterinary Institute (NVI) of Ethiopia.

### Serological assay

#### Rose bengal plate test (RBPT)

Rose Bengal Plate Test is simple, rapid test used for screening animal brucellosis. It was carried out in ISO/IEC 17025:2017 accredited laboratory, and ready to use *Brucella abortus* antigen for RBT was obtained through purchased (ID Vet, France). Rose Bengal Antigen lot 275/23 concentrated *B. abortus* suspended in buffered diluents and stained with Rose Bengal dye was used. Briefly, 30 µl of Brucella antigen was mixed with an equal amount of serum on a plate card with a single tip and spread onto entire circles about 2 cm in diameter, according to the manufacturer’s enclosed instructions. Cards were manually rotated for four minutes according to the standard procedure described in OIE [28]. Any degree of agglutination or clumping is considered positive, while the absence of clumping or agglutination is considered negative.

#### Indirect enzyme-linked immunosorbent assay (I-ELISA)

I-ELISA is an immunological assay used to detect Brucella antibodies directed against *B. abortus*, *B. melitensis*, and *B. suis* LPS antigen [28]. Briefly, all sera samples, controls, and reagents were brought to room temperature (18–25ºC). Diluted sera samples (1/20) to be tested and controls were incubated at room temperature for 45 min with *B. abortus* Lipopoly saccharide (LPS) coated plates. Following washing,100µL multispecies Horseradish Peroxidase (HRP) conjugate was dispensed to each microplate well and kept at room temperature for thirty minutes. After washing to remove excess conjugate, a Tetra methyl Benzidine (TMB) substrate solution was dispensed and kept in a dark place for fifteen minutes. The resulting color developed is depending on the amount of specific antibody present in the sample to be tested. Finally, plates were read using an ELISA microplate reader with a 450 nm wavelength (Lab system, USA). The percent value (S/P%) was calculated using the formula recommended by the kit manufacturer (ID Vet, France) for lot BRUS –MS ver 1014GB kit protocol.

#### Complement fixation test (CFT)

A complement fixation test is a classical laboratory diagnostic test used to detect the presence of Brucella antibodies against Brucella antigens in sheep, goats and cattle sera [28]. Veronal buffer ingredients for CFT were obtained through purchased (ID Vet, France). The National Veterinary Institute Research and Development Laboratory lot numbers 01/15, 01/13, 03/18, and 03/18 were used to obtain guinea pig complement, hemolytic serum, negative control, and positive control, respectively. A complement fixation test was conducted in accordance with the procedure described in OIE [28]. Undiluted test sera were inactivated in a water bath at 60 °C for 30 min. In each row of 96 wells plates, 25 µL inactivated and 1/5 diluted test sera were dispensed. Twenty-five µL antigen diluted to working strength (1/10) was added to the wells in rows B, D, F, and H, and a similar volume of veronal buffer was dispensed in the anti-complementary rows (A, C, E, and G). The antigen-test sera mixture and all control wells were incubated at 37 °C for 30 min. Followed by 25µL working complement of checked strength was dispensed to each well and incubated at 37 °C for 30 min. All control wells were handled in separate plate. Then, in each well, a 1:1000 diluted 25µL hemolytic serum in 1% Sheep Red Blood Cell (SRBC) was also dispensed. The plates were re-heated at 37 °C for 30 min. Plates were kept at 4 °C for 2–3 h to allow unlysed cells to settle before reading the results. The percent lyses were evaluated against anti-complementarity wells. RBC settlement greater or equal to 50% is considered positive results, and greater or equal to 50% lyses are considered negative results [28].

#### PCR detection

**Table 1:** Primer pairs used for the detection of *Brucella* genus and *Brucella* species

Target pathogen	Primer	Sequences	Amplified Products	References
Brucella, BCSP31	B4 (F)	5ʹ-TGGCTCGGTTGCCAATATCAA-3ʹ	223 bp	[29]
	B5 (R)	5ʹ-CGCGCTTGCCTTTCAGGTCTG-3ʹ		
*B. abortus* BA	F	5ʹ-CCATTGAAGTCTGGCGAGC-3ʹ	156 bp	[30]
	R	5ʹ-CGATGCGAGAAAACATTGACCG-3ʹ		
*B. melitensis* IS711	F	5ʹ-TGCCGATCACTTAAGGGCCTTCAT-3ʹ	731 bp	[31]
	R	5ʹ-AAA TCGCGTCCTTGCTGGTCTGA-3ʹ		

The *Brucella* genome was extracted using a commercially available tissue and blood extraction kits (Qiagen, Germany). Thirty sero-positive sera samples from three serological tests (1 sheep, 11 goats, and 18 cattle, with 17 sheep and 7 goats CFT negative sera) were subjected to PCR amplification using Brucella genus-specific, *B. abortus*, and *B. melitensis* species-specific primers. The primers listed at Table 1 were used for PCR assay. They are expected to amplify a 223 bp sequence of *Brucella* genus-specific genome; 156 bp *B. abortus* and 731 bp *B. melitensis* [29].

A total of 25µL PCR reaction volume was used. The reaction mixture include: forward and reverse primers at a concentration of 5pmol/µL each 2µL, 1xPCR reaction buffer 4.5µL, Dream Taq polymerase 5U 1.5µL, 2mMol each dNTPs 5µL, 10x Dream Taq buffer containing 20mMol MgCl_2,_ 5µL (Thermo Scientific, USA) and template 5µL of DNA. DNA extracted from *Brucella abortus* which is conserved in all *Brucella* species and known extracts of *Brucella abortus* and *Brucella melitensis* DNA antigen were used as positive control. The PCR reaction conducted using a Thermocycler (Applied biosystem 2720, Singapore). PCR amplification were carried out as initial denaturation of 95 °C for 5 min, followed by 40 cycles of 95 °C for 1 min denaturation, annealing at 62 °C for 30 s, and extension 72 °C for 30 s, and a final elongation step of 72 °C for 5 min [29]. In order to check the presence or absence of specific PCR product bands, 10 µL PCR amplicon were run in a 2% w/v agarose gel electrophoresis and then visualized under an Ultra Violet transilluminator (UVtec, France).

### Data analysis

Microsoft Excel spreadsheet program was used for data storage and data were analyzed using the Statistical Package for Social Science (SPSS) version 21 software program [32]. Sero-prevalence in sheep, goats and cattle were computed by the ratio of RBPT, I-ELSA, CFT, and PCR Positive animals to the total number of animal’s sera tested. The cross-tabulated 2 × 2 contingency table was used to analyze true positive (TP), true negative (TN), false positive (FP), and false negative (FN), and sensitivity and specificity were determined using Receiver Operating Characteristics curve analysis software [32].

## Results

### Serological test result

All three serological test methods revealed that cattle had the highest brucellosis prevalence among the 2,317 sera samples tested. Among the samples tested for RBPT, I-ELISA, and CFT, 189 (8.2%) tested positive for RBPT, 191 (8.2%) tested positive for I-ELISA, and 48 (2.1%) tested positive for CFT (Table 2).

**Table 2:** Prevalence of brucellosis in different animal species

Animal species	No tested	RBPT positive	I-ELISA positive	CFT positive	PCR positive
Sheep	552	25 (4.5%)	18 (3.3%)	1 (0.2%)	17/18 (94.4%)
Goats	1345	114 (8.5%)	105 (7.8%)	11 (0.8%)	18/18 (100%)
Cattle	420	50 (11.9%)	68 (16.2%)	36 (8.6%)	18/18 (100%)
Total	2317	189 (8.2%)	191 (8.2%)	48 (2.1%)	53/54 (98.2%)

### PCR detection result

**Fig. 1:**
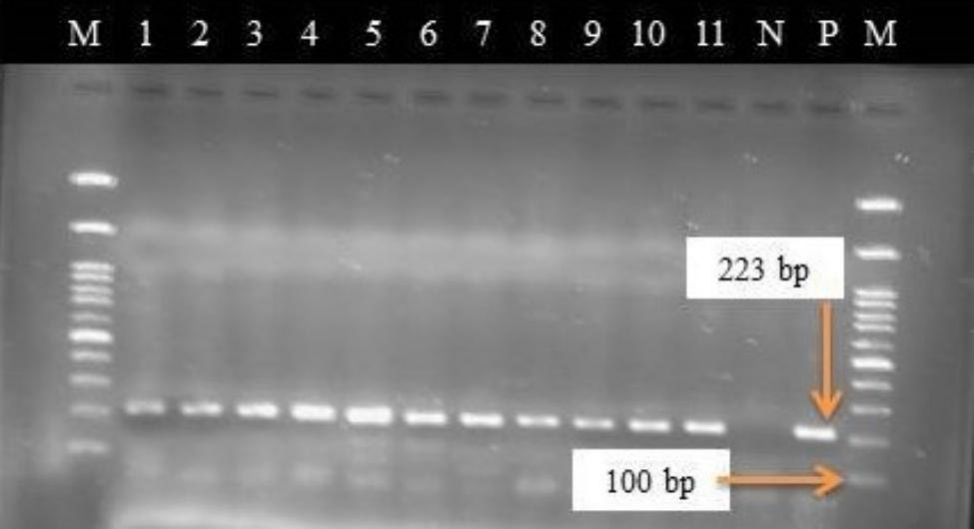
Electrophoresis picture showing a 223 bp Brucella genus-specific PCR product from 11 representative sera samples on 2% agarose gel. The first and last lane: Molecular ladder (100 bp plus, Fermentas), Lanes 1–4: Cattle sera, Lanes 5–8: Sheep sera, Lanes 9–11: Goats sera, Lanes N and P: negative and positive controls, respectively

**Fig. 2:**
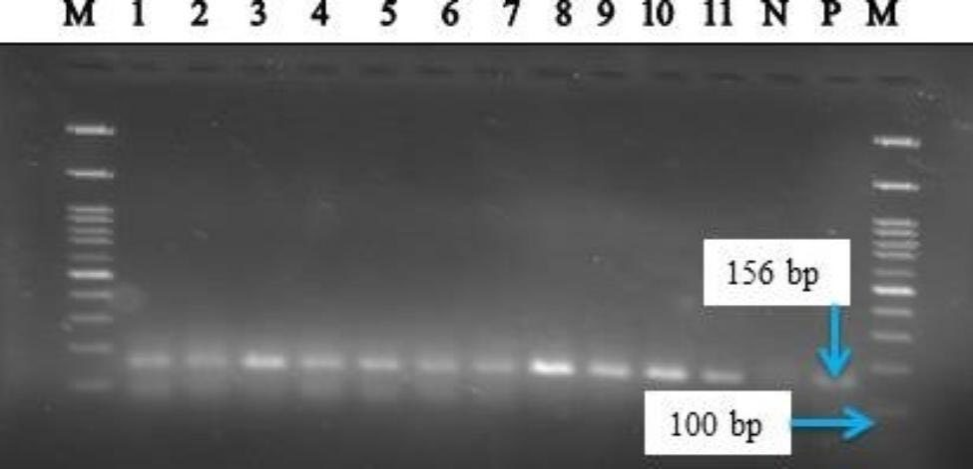
Electrophoresis analysis of *B. abortus* specific PCR products (156 bp) on 2% agarose gel. The first and last lane: Molecular ladder (100 bp plus, Fermentas), Lanes 1–4: Cattle sera, Lanes 5–8: Sheep sera, Lanes 9–11: Goats sera, Lanes N and P: negative and positive controls, respectively

Thirty sero-positive sera samples for all three serological tests (1 Sheep, 11 Goats, and 18 cattle), 30/30 (100%) were also positive by PCR. Interestingly, out of 17 sheep sera, 16 (94.12%) and 7 goat sera (100%) samples that showed negative result by CFT, 23 (95.83%) were positive when analyzed by PCR (Figs. 1 and 2).

## Discussion

Brucellosis is a threat to both human and animal health. Brucella infection persists in an environment where disease control is not given much consideration. In Ethiopia, numerous findings for the existence of brucellosis in ruminants, particularly cattle brucellosis, have been reported in various regions. However, the specificity and sensitivity of existing serological tests data are limited, with the exception a single report on bovine brucellosis published by the National Animal Health Diagnostic and Investigation Center [33]. Sensitive and specific tests allow us to reduce diagnostic disparity in discriminating between infected and non-infected individual, as well as avoid unnecessary casualties when the tests incorrectly categorize the animals [34]. In this study, the specificity and sensitivity of tests were evaluated using Receiver Operating Characteristics (ROC) curve analysis software, SPSS [32]. The agreement of the tests is determined by the area under the curve (AUC): A score of 1 indicates perfect agreement, a score of 0.8 to 0.9 is very good agreement, a score of 0.7 to 0.8 is good, a score of 0.6 to 0.7 is fair, a score of 0.5 to 0.6 is poor agreement, and a score of less than 0.5 indicates no agreement between tests [35]. The I-ELISA test was chosen as the reference test when determining the specificity and sensitivity of RBPT and CFT tests. As a result, excellent agreement was found between RBPT and I-ELISA in sheep and goat (0.983 and 0.998, respectively), and very good agreement was found in cattle (0.868). The test result comparisons between CFT and I-ELISA, on the other hand, revealed good agreement in cattle (0.765) but poor agreement in sheep and goat (0.529 and 0.552, respectively).

The RBTs test and plate card reading is simple, quick, and sensitive enough to detect all brucellosis infected sheep and goats. However, I-ELISA detected a false positive in 7/552 (1.3%) sheep and 9/1345 (0.7%) goats. Unlikely, 18/420 (4.3%) cattle sera were detected by I-ELISA but were negative for RBPT, indicating a false negative reaction (Table 3**)**. As a result, RBPT positive samples were subjected to reliable, specific tests to ensure a conclusive diagnosis. I-ELISA provided excellent specificity and sensitivity while maintaining reproducible, simple to conduct with minimal equipment, and kits were easily accessible from a saleable source. Padilla et al. [36] described that ELISAs are more appropriate than CFT for use in less equipped laboratories, and ELISA facilities are now used to detect a variety animal and human diseases [37]. The Specificity of CFT was found to be excellent, but its sensitivity was the lowest compared to RBPT and I-ELISA. This could be due to samples hit or terminate complement provoking action in the assay even in the absence of antigen. Such “anti- complementary” activities result in a test void and the inability to find CFT results [38].

**Table 3:** Determining the specificity and sensitivity of RBPT where I-ELISA as reference test

Host species		I-ELISA +	I-ELISA-	Total
Sheep	RBPT+	18	7	25
	RBPT-	-	527	527
	Total	**18**	**534**	**552**
Goats	RBPT+	105	9	114
	RBPT-	-	1231	1231
	Total	**105**	**1240**	**1345**
Cattle	RBPT+	50	-	50
	RBPT-	18	352	370
	Total	**68**	**352**	**420**

Using I-ELISA as the reference test, the sensitivity of RBPT in sheep, goats, and cattle was 100% (95% CI: 98.8–99.8), 100% (95% CI: 99.4–99.9), and 74% (95% CI: 80.4–93.1), respectively. Specificity for RBPT in sheep, goats and cattle was 98.69% (95% CI: 98.8–99.9), 99.28% (95% CI: 99.4–99.9), 100% (95% CI: 99.9-100), respectively. In this study, RBPT was found to be sensitive enough to detect 100% brucellosis infected sheep and goats. This report is in agreement with Pappas et al. [39], who reported RBPT is highly sensitive (> 99%). The current study found a sensitivity of 74% and a specificity of 100% for RBPT in cattle, which is lower in sensitivity and higher in specificity than Getachew et al. [33], who found a specificity of 84.5% and a sensitivity of 89.6% for RBPT in cattle. Our RBPT produced some false positive results in sheep and goats that were not detected by I-ELISA. This finding is supported by another study, suggested that RBPT has a limitation in distinguishing agglutinating reactions caused by *Brucella* infection from those caused by cross-reacting bacteria such as *Yersinia enterocolitica* O:9 [17].

Other authors have described lipopolysaccharide molecules with similar antigenic epitopes that cross-react with a variety of Gram-negative bacteria, including: *Salmonella enterica* serovar, *Escherichia coli* O116, and O157, *Vibrio cholerae* [39, 40]. We found that eighteen I-ELISA positive cattle sera were negative by RBPT, which could be attributed to the prozone phenomenon. According to Padilla et al. [36], the prozoning effect causes excess overlapping antibodies in the assay and false-negative reactions. In the current study, the sensitivity and specificity of RBPT in goats were 100% and 99.27%, respectively, which is in consistent with the previous finding reported by Muktajeul et al. [25] reported 100% sensitivity and 96.22% specificity. The sensitivity in sheep was 100% and specificity 98.69%, which is slightly higher than the previous report by Mahajan et al. [24], which was 87% and 95%, respectively.

The highest specificity and sensitivity was observed in I-ELISA. The high accuracy of I-ELISA may be due to its ability to identify minute amounts of antibodies present in the initial course of infection, which RBPT does not. Our diagnostic sensitivity of I-ELISA was 100%, which was higher that RBPT and CFT but similar specificity with CFT (100%). This is slightly higher than the sensitivity of 96.8% and specificity of 96.3% reported by Getachew T. et al. [33] for I-ELISA in cattle. Paweska et al. [26] reported 99.8% specificity and 100% sensitivity when 4803 cattle sera were tested against I-ELISA. And 100% agreement with [38] who found 100% sensitivity in cattle. According to the findings of this study, the I-ELISA assay is highly specific (100%) in all three species and the most sensitive diagnostic test for cattle brucellosis. The ability to detect antibodies of all isotopes may account for this accuracy [36].

Chand et al. [37] recommended that ELISA is superior to RBPT for determining the condition of brucellosis in cattle because the detection ability of an infected animal in ELISA is higher. Erdenebaatar et al. [27] stated that I-ELISA can be used to avoid nonspecific reactors in RBPT positive sera. In the late stages of the disease, non-agglutinating antibodies become more abundant than clumping antibodies, potentially leading to non-detection. The incorporation of IgG conjugate in I-ELISA allows for the detection of these non-clumping antibodies. Using RBPT in conjunction with ELISA allows us to identify all the positive Brucella reactors. In our findings, we avoided false-negative results in cattle and identified potential false-positive reactors in sheep and goats. This is covenant with previous results reported by Padilla et al. [36], who determined that detecting all Brucella reactors with a single agglutination test was impossible. The combination of RBPT with ELISA is recommended since the ELISA test is reproducible and reliable.

**Table 4:** Determining sensitivity and specificity of CFT where I-ELISA as the reference test

Host species		I -ELISA +	I-ELISA -	Total
Sheep	CFT+	1	-	1
	CFT-	17	534	551
	Total	**18**	**534**	**552**
Goats	CFT+	11	-	11
	CFT-	94	1240	1334
	Total	**105**	**1240**	**1345**
Cattle	CFT+	36	-	36
	CFT-	32	352	384
	Total	**68**	**352**	**420**

Considering I-ELISA as a reference test, the sensitivity and specificity of CFT were 5.56% and 100% in sheep, 10.48% and 100% in goats, and 52.94% and 100% in cattle, respectively (Table 4**)**. The current comparative serological study revealed that the CFT had the highest specificity in all three species sera but the lowest sensitivity. This could be due to the presence of anti-complementary effect, as reagents and complement may not be fixed by many classes of antibodies in animals. Padilla et al. [36] explained this as the result of only the Immunoglobulin G1 isotope of antibody binding complement well, increasing test specificity. Other limitations include bias in reading results, destroying complement activator (anti-complementary activity), and the test’s inability to be used with hemolysed serum specimen, which limits its sensitivity.

Among molecular techniques, PCR has been described as a device to allow fast and more sensitive identification of Brucella DNA from serum samples [29]. It has been regarded as the preference test for the identification of Brucella species as it had a greater specificity and sensitivity for detection when compared to other immunological or phenotypic techniques [40]. Mitka et al. [41] conducted four different PCR assays. All four techniques demonstrated 100% sensitivity and specificity, making them suitable for detecting both acute and relapsing brucellosis in humans. In line with these findings, the results of our study revealed that the conventional PCR test was capable of detecting *Brucella* DNA from CFT negative sheep and goat sera as well as all cattle sero-positive samples. Thirty sera (1 sheep, 11 goats, and 18 cattle) were found to be sero-positive for all three serological tests, with 30/30 (100%) also showing positive PCR results. Interestingly, out of 17 sheep sera, 16 (94.12%) and 7 goat sera (100%) samples that showed negative results by CFT, 23 (95.83%) were positive by PCR. Only one I-ELISA positive sheep serum sample was not detected by PCR. This finding is consistent with [22], who stated that PCR has a lower detection ability than ELISA. According to [22], the detection ability of PCR techniques is dependent on the DNA extraction method and exposure to inhibitors such as phenol, DNAse, and EDTA.

According to the PCR assay used in this study, all sero-positive samples from cattle, sheep, and goats in serological tests were found to be positive for *B. abortus*, but *B. melitensis* was not detected in either ruminant species. This finding is consistent with the findings of [30], who investigated 1270 goat and 770 sheep sera samples. By real-time PCR, all sero-positive sera samples from these small ruminants were found to be positive for *B. abortus*, but *B. melitensis* was not identified. The authors hypothesized that inter-species transmission could be caused by mixed farming, in which small and large ruminants share the same grassland. *B. abortus* may be problematic in non-preferred hosts, such as small ruminants. According to the same authors, Brucella’s eco-diversity and multi-pathogenicity allow it to cross the species barrier [30].

## Conclusion and recommendation

In this study I-ELISA was found to be the most specific and sensitive test. RBPT was also sensitive enough to detect all brucellosis-infected sheep and goats. RBPT, on the other hand, produced false positive results in sheep and goats and false negative results in cattle. Therefore, RBPT should be used in conjunction with I-ELISA to address issues related to RBPT sensitivity and specificity. This study’s PCR assay confirmed the presence of *Brucella abortus* in small and large ruminants consistent with the study areas. This is the first report in Ethiopia on the detection of *B. abortus* in small ruminant serum. *B. abortus* was found in non-preferred hosts. Due to resource limitation we couldn’t confirm all sero-positive sera with PCR. We recommend further in depth research is needed to determine the genetic diversity of *B. abortus* and other Brucella species, as well as to understand their molecular epidemiology at national scale, in order to design cost-effective preventive and control measures.

## Data Availability

All data analyzed during this study are included in the manuscript. However, the raw data is available from the corresponding author upon formal request.

## Abbreviations

CFT: Complement Fixation Test
DNA: Deoxyribose Nucleic Acid
I-ELISA: Indirect Enzyme Linked Immunosorbent Assay
NVI: National Veterinary Institute
RBPT: rose Bengal plate test
PCR: polymerase chain reaction
TMB: Tetra Methyl Benzidine
SRBC: Sheep Red Blood Cell
LPS: Lipopolysaccharide
SPSS: Statistical Package for Social Sciences. WOAH:World Organization for Animal Health. OIE:International Organization for animal Epizootic
WOAH: World Organization for Animal Health
OIE: International Organization for animal Epizootic
